# The role of family planning in achieving safe pregnancy for serodiscordant couples: commentary from the United States government’s interagency task force on family planning and HIV service integration

**DOI:** 10.7448/IAS.20.2.21312

**Published:** 2017-03-08

**Authors:** Jennifer Mason, Amy Medley, Sarah Yeiser, Vienna R. Nightingale, Nithya Mani, Tabitha Sripipatana, Andrew Abutu, Beverly Johnston, D. Heather Watts

**Affiliations:** ^a^ Office of Population and Reproductive Health, U.S. Agency for International Development, Arlington, VA, USA; ^b^ Division of Global HIV and AIDS, U.S. Centers for Disease Control and Prevention, Atlanta, GA, USA; ^c^ Office of HIV/AIDS, U.S. Agency for International Development, Arlington, VA, USA; ^d^ HIV/AIDS Prevention Program, U.S Department of Defense, Falls Church, VA, USA; ^e^ Office of the U.S. Global AIDS Coordinator, U.S. Department of State, Washington, DC, USA

**Keywords:** Family planning, HIV, serodiscordant couples, safer pregnancy, United States Government, integration

## Abstract

**Introduction:** People living with HIV (PLHIV) have the right to exercise voluntary choices about their health, including their reproductive health. This commentary discusses the integral role that family planning (FP) plays in helping PLHIV, including those in serodiscordant relationships, achieve conception safely. The United States (US) President’s Emergency Plan for AIDS Relief (PEPFAR) is committed to meeting the reproductive health needs of PLHIV by improving their access to voluntary FP counselling and services, including prevention of unintended pregnancy and counselling for safer conception.

**Discussion:** Inclusion of preconception care and counselling (PCC) as part of routine HIV services is critical to preventing unintended pregnancies and perinatal infections among PLHIV. PLHIV not desiring a current pregnancy should be provided with information and counselling on all available FP methods and then either given the method onsite or through a facilitated referral process. PLHIV, who desire children should be offered risk reduction counselling, support for HIV status disclosure and partner testing, information on safer conception options to reduce the risk of HIV transmission to the partner and the importance of adhering to antiretroviral treatment during pregnancy and breastfeeding to reduce the risk of vertical transmission to the infant. Integration of PCC, HIV and FP services at the same location is recommended to improve access to these services for PLHIV. Other considerations to be addressed include the social and structural context, the health system capacity to offer these services, and stigma and discrimination of providers.

**Conclusion:** Evaluation of innovative service delivery models for delivering PCC services is needed, including provision in community-based settings. The US Government will continue to partner with local organizations, Ministries of Health, the private sector, civil society, multilateral and bilateral donors, and other key stakeholders to strengthen both the policy and programme environment to ensure that all PLHIV and serodiscordant couples have access to FP services, including prevention of unintended pregnancy and safer conception counselling.

## Introduction

The United States (US) President’s Emergency Plan for AIDS Relief (PEPFAR) is the US Government (USG) initiative to help save lives of those suffering from HIV/AIDS around the world [1]. The USG, including PEPFAR, supports a person’s right to choose, as a matter of principle, the number, timing and spacing of their children, as well as the voluntary use of family planning (FP) methods, regardless of HIV status [2]. The USG PEPFAR Interagency Task Force on FP/HIV integration is comprised of FP and HIV experts from USG developmental agencies who provide technical guidance to PEPFAR policies and programmes on integrating FP and HIV services. The Task Force previously explained the USG principles and the continued commitment and efforts in meeting the FP needs of people living with HIV (PLHIV) in a 2013 commentary [3].

PEPFAR supports the UNAIDS targets of 90–90–90 to end the AIDS epidemic [4] and has moved to focus its programme investments in an effort to contribute to these targets. These goals state that by 2020, 90% of all PLHIV will know their HIV status; 90% of all people with diagnosed HIV infection will receive sustained antiretroviral therapy (ART) and 90% of all people receiving ART will achieve viral suppression. Bidirectional integration of voluntary FP and HIV services can improve the health and quality of life of people seeking FP services while making progress towards reaching these global targets. With the global push to reach 90–90–90 and the release of the World Health Organization (WHO) guidelines on Test and Start and pre-exposure prophylaxis (PrEP), there are new opportunities to further integrate FP and HIV services [5]. PEPFAR is committed to meeting the sexual and reproductive health needs of PLHIV by improving their access to voluntary FP counselling and services, including unintended pregnancy prevention and safer conception counselling, through integration of FP services into HIV prevention, care and treatment programmes.

PLHIV have the right to exercise voluntary choices about their health, including their reproductive health. Those who wish to have children should have access to safer conception and pregnancy counselling services. FP is an integral tool to help all women, including those living with HIV (WLHIV) and in serodiscordant relationships, achieve safer conception and improve pregnancy outcomes, by enabling women to delay pregnancy until she and/or her partner is healthy enough and personally ready for pregnancy. Integration of FP services in HIV care and treatment settings allows healthcare providers to counsel both women and men on FP according to their fertility intentions, HIV status and treatment regimen and helps ensure that PLHIV have access to FP services that support their fertility choices in a safe and nonjudgemental environment. WLHIV and their partners should be engaged in the development, implementation and monitoring of FP services to ensure services address their needs and respect their rights.

## Discussion

### Implementing preconception care for PLHIV

Fertility intentions should be discussed on a regular basis during the routine medical care offered to PLHIV in all clinical and community settings. While infertility is common, affecting an estimated one in every four couples, infertility treatment (e.g. clomiphene, hormone injections, tubal surgery, *in vitro* fertilization etc.) remains mostly inaccessible in resource-limited settings [6]. Women and couples reporting issues with infertility should be referred to fertility services, where available. Further discussion of infertility is beyond the scope of this commentary, which focuses on preconception services for PLHIV.

The goal of preconception care and counselling (PCC) for PLHIV is to ensure that both men and women are in optimal health before pregnancy, that every pregnancy is planned and that the risk of HIV transmission to the partner (through sexual transmission) and child (through pregnancy, delivery or breastfeeding) has been minimized. This includes ensuring that WLHIV are stable on ART and have achieved viral suppression prior to conception as evidence suggests that the risk of perinatal transmission is virtually eliminated if women achieve viral suppression before conception [7]. Inclusion of PCC, including comprehensive information and counselling on all available FP methods, as part of routine HIV services is essential to prevent unintended pregnancies among PLHIV and perinatal infections [8,9]. Table 1 outlines strategies for implementing preconception care for PLHIV [8,10–18].

**Table 1.** Integration of family planning into preconception care (PCC) for people living with HIV (PLHIV).

**Where should PCC services be offered?**	**Where should FP services be integrated into PCC?**
Integrated into all services targeting PLHIV (e.g. ART, care and support, ANC/PMTCT) Offered in both facility and community settings	FP should be integrated into all HIV services, including PCC in facility and community settings FP services should be offered to all clients/couples who express interest in PCC services and/or in delaying or limiting pregnancies The scope of FP services that should be offered depends upon the client/couple’s interest in using an FP method. Ideally, FP education, counselling and method provision should be available at all sites where ART and PCC services are provided
**Who should provide PCC services?**	**Who should provide FP services for PLHIV and serodiscordant couples?**
Healthcare providers (i.e. doctors, nurses, midwives) who routinely deliver services to PLHIV in facility and community settings Task sharing some services with community health workers and lay counsellors can help address human resource constraints	Healthcare providers (i.e. doctors, nurses, midwives) who routinely deliver ART care and treatment and PCC services to PLHIV in facility and community settings Task sharing some services with community health workers and lay counsellors can help address human resource constraints If FP services are not available or are limited in HIV settings, clients should be referred to health platforms that offer comprehensive FP services by trained MCH/FP providers
**When should PCC services be offered?**	**FP service provision in PCC services**
Initially offered to all women, men and couples living with HIV of childbearing age during first few visits after HIV diagnosis and ART initiation Offered at least annually thereafter After a change in relationship status (e.g. new sex partner) Client expresses a desire to obtain or avoid pregnancy during routine clinical exam	Voluntary FP services should be offered to female and male PLHIV and serodiscordant couples at all HIV testing, care and treatment visits PLHIV and serodiscordant couples should always be informed that FP services are voluntary and that ART or PCC services are not contingent upon use of FP services Comprehensive FP services including education, counselling, method provision and follow-up care should be provided at the same site as HIV services; however, if a full spectrum of FP services cannot be provided, clients should receive facilitated referrals to an appropriate health setting The scope of FP services provided to each client should depend upon their fertility intentions, interest in FP and history/current use of FP Women/Couples who are using an FP method should receive follow-up FP services at regular HIV visits, including discussion of fertility intentions, medical updates, condom provision, oral contraceptive refills, injections, IUD checks and so on. They should be assessed for their satisfaction with the contraceptive and provided with counselling for management of side effects or guidance for switching methods, if desired Women/Couples who are not using an FP method who do not wish to become pregnant immediately should receive FP education on the benefits of FP and types of FP methods available, as well as general information on medical eligibility, effectiveness and duration of available FP methods. This type of education can be done in a group or through interpersonal communication in a facility or community setting If the woman is interested in using an FP method, she should receive comprehensible counselling on the contraceptive chosen that includes information on how the method works/is applied, possible side effects, duration of efficacy, danger signs and instructions for follow-up visits. If the woman is medically eligible to use the desired method, it should be provided to her on site or through facilitated referral For international development activities, the 5th Edition of the WHO Medical Eligibility Criteria for Contraceptives should be used by health providers to assist women make informed decisions regarding the best FP method for their individual situation, which should address their health status, fertility intentions, social and medical preferences and SRH-related behaviours
**Key elements of PCC services**	**Key elements of FP activities within PCC services**
Assess fertility intentions, if client is unsure of fertility intentions or plans to have children, provide PCC services, including FP information and counselling to the client and partner***Services for women and men who are considering a pregnancy soon*** Counsel on risk factors for HIV/STI transmission and acquisition and strategies for reducing this risk Provide support for HIV serostatus disclosure, partner involvement and partner HIV testing Assess and counsel about timing and spacing of pregnancy to optimize maternal health (e.g. adherent and stable on ART) and infant outcomes Offer voluntary FP education and counselling to optimize pregnancy timing Screen for and manage other co-morbidities (e.g. mental health, non-communicable diseases and infectious diseases) Screen for and counsel about the need to eliminate or reduce substance use (e.g. alcohol, cigarettes) Provide assessment, treatment and partner management of other STIs Conduct a medication history to ensure no drug–drug interactions or medications contraindicated during pregnancy Discuss safer conception options (e.g. ART for positive partner(s), PrEP for negative partner, limited and timed condom-less sex and self-insemination) Screen for and refer for infertility and subfertility management Screen for nutritional deficiencies/disorders and offer food supplemental and fortification Review vaccination history and provide immunizations as needed***Services for women and men who wish to delay or limit pregnancy*** Provide safer pregnancy counselling for clients who wish to have children in the future or are unsure of their fertility intentions Provide voluntary FP education and counselling Provide FP methods either onsite or through facilitated referral	Assess fertility intentions, if client is unsure of fertility intentions or plans to have children, provide PCC services, including FP information and counselling to the client and partner***FP services for women and men who are considering a pregnancy soon*** Ensure that client/couple understands that FP use is voluntary and that PCC services are not contingent upon use of FP. Provide FP education and counselling to PLHIV and serodiscordant partner to support optimal timing of pregnancy. Include education on dual method use to prevent both STIs and pregnancy Offer FP methods that have a very short return to fertility period, such as condoms or other barrier methods, oral contraceptives or the copper IUD either onsite or through facilitated referrals Provide client/couple information and/or referrals for post-partum FP See “FP service provision in PCC services” for more detail on services that should be offered***FP services for women and men who wish to delay or limit pregnancy*** Ensure that client/couple understands that FP use is voluntary and that ART or PCC services are not contingent upon use of FP Provide FP education and counselling to PLHIV and serodiscordant partner to support their desire to delay or limit pregnancy Provide information on dual method use to help prevent both STIs and pregnancy Provide information about potential interactions between hormonal contraceptive methods and certain ARVs, especially on the potential decreased efficacy of hormonal implants if the client is using an efavirenz containing regimen Provide information on hormonal contraception and potential HIV acquisition risk to clients in serodiscordant relationships Offer a wide range of FP methods including short and long-acting contraception either onsite or through facilitated referrals If desired, provide counselling and referrals for permanent FP methods See “FP service provision in PCC services” for more detail on services that should be offered
**How should preconception care services be offered?**	**How should FP services be offered as part of PCC?**
As much as possible, PCC services should be offered under one roof to maximize PLHIV’s access to these services. Facilitated referral systems, with feedback and documentation, should be developed and implemented for all services not offered onsite Whenever possible, PCC services should be offered to the couple in order to provide an opportunity for shared counselling and FP decision-making Further research needed to determine innovative service delivery models, including community provision	FP services should be offered where and when PCC services are offered, with comprehensive integrated services as the ideal. When comprehensive FP services cannot be provided alongside PCC services, the client/couple should be offered facilitated referrals to obtain desired FP services HIV/PCC providers should be trained and supported to provide high quality, non-judgemental FP services, as part of efforts to provide integrated SRH services to PLHIV When appropriate, FP couples counselling should be provided to increase communication and understanding and support of FP More information on models for integrating FP into PCC services is needed, including tools for monitoring quality of care

### When and where should PCC-including FP services be offered?

PCC services should be offered in both facility and community settings to improve PLHIV’s access to these services. Healthcare providers should proactively discuss childbearing intentions with their clients living with HIV. Clients who do not wish to become immediately pregnant, including those who are unsure of whether they want any/more children, should be provided with FP and safer conception and pregnancy counselling and offered a wide range of FP methods, including short and long-acting contraception. Because the decision to have a child may change over time, childbearing intentions of both male and female clients should be assessed during ART initiation and routine HIV care and treatment services [10,11] as well as during other health encounters including post-partum care, post-abortion care and non-communicable disease visits. PCC services should also be offered to PLHIV who report a change in their fertility intention during the clinical encounter [8]. To expand awareness of PCC and improve utilization of PCC and FP services, health providers should discuss fertility intentions with male PLHIV who have female sex partners and offer FP and safer conception and pregnancy counselling to them and their partners.

### Services for PLHIV who are considering a pregnancy soon

Safer conception counselling should be offered to PLHIV who are considering a pregnancy soon. For PLHIV who desire children, partner testing and safer conception and pregnancy counselling are critical to reduce the risk of HIV transmission to HIV-negative partners. Safer conception counselling includes education about the risk factors for transmitting HIV to their partner(s) and infant and strategies (see Table 1) for mitigating those risks. Safer conception practices can reinforce adherence and retention to ART, leading to HIV treatment optimization. Providers should determine if the partner’s HIV status is known and whether the HIV status of the HIV-positive partner has been disclosed. If not, support for HIV serostatus disclosure should be provided to encourage communication about HIV risks and future childbearing to enable the couple to support each other in adhering to HIV treatment regimens and other medical advice [8,10]. Partner and couples HIV testing services should also be offered to allow tailoring of messages and services according to the couple’s HIV status. Women who test HIV-negative at the initial visit should be offered to repeat HIV testing during the third trimester and breastfeeding period to reduce the elevated risk of vertical transmission associated with seroconversion [19]. Routine assessment, treatment and partner management of other sexually transmitted infections (STIs) should also be offered as many STIs can have harmful effects on pregnant women and their infants and can reduce fertility in both men and women [13].

Providers should discuss safer conception options that minimize the risk of HIV and STI transmission. FP use in conjunction with ART for the HIV-positive partner and peri-conception PrEP for the HIV-negative partner provides couples with effective tools to time their pregnancies and reduce the risk of HIV transmission to the seronegative partner and to the infant [20]. FP methods that do not have a prolonged return to fertility period may assist couples in avoiding pregnancy for a short duration until optimal health and reduced risk for transmission is achieved. Ensuring that the HIV-positive partner is stable on ART and has achieved viral suppression can reduce the risk of HIV transmission during conception by 96% [21,22]. PrEP is an additional safer conception strategy for couples where the HIV-positive partner has not yet achieved viral suppression, is not yet on ART or where the partner’s HIV status is unknown [23–25]. PrEP includes daily dosing of a combination of antiretrovirals (ARVs) in HIV-negative individuals; studies in discordant couples have demonstrated reductions of 63–75% in HIV acquisition risk [26,27]. Safety data from clinical trials indicate no increased risk of harm to the pregnancy itself or infant growth when PrEP is used during the peri-conception period. The impact on infant growth among infants exposed to PrEP in utero remains incomplete. Women should have the opportunity to weigh the risks of vertical transmission associated with acute infection against the possible benefits of PrEP use, especially in cases where the HIV status of the partner is unknown [28]. Non-ART based safer conception strategies include timed condom-less intercourse during the peri-ovulation period when the woman is most fertile, with condom use at all other times, and self-insemination with a negative partner’s sperm during the peri-ovulatory period [29–31].

### Services for PLHIV who would like to delay or limit pregnancy

PLHIV or serodiscordant couples who wish to delay pregnancy should be provided with FP and safer pregnancy counselling along with access to a broad range of FP methods, including short and long-acting contraception. Female and male PLHIV who do not wish to have children/more children may choose to receive counselling and referrals for permanent FP options. FP education and counselling for WLHIV or women in serodiscordant relationships should mirror that of standard FP services; including counselling on dual method use [14]; however, provision of additional information on the WHO Medical Eligibility Criteria guidance for hormonal contraception (HC)-HIV acquisition and HC-ART interactions is essential [15]. According to the WHO, WLHIV and women at high risk of acquiring HIV can safely use all available FP methods, including HC and intrauterine devices [32]. Dual method use (condoms plus a highly effective FP method) can help prevent transmission and acquisition of other STIs or more virulent or resistant strains of HIV and unintended pregnancies [32–34]. Providers should be aware of potential interactions between hormonal contraceptives and certain ARVs that may lower contraceptive efficacy and counsel their clients appropriately. The evidence to date for ARV-associated contraceptive failure leading to pregnancy has only been linked to drug interactions between efavirenz and the levonorgestrel-releasing Jadelle implant. However, decreased blood levels of hormone with Implanon use remains a concern and requires further monitoring. Currently, there is no evidence linking contraceptive method failure rates with non-efavirenz-containing ART regimens. There are data on other ARVs including nevirapine and lopinavir/ritonavir that are reassuring, but additional data are needed for other regimens. Despite an apparent decrease in contraceptive efficacy among WLHIV using implants and an efavirenz-containing ART regimen, the effectiveness remains very high, especially in comparison with other shorter acting hormonal methods [35–41].

Providers should also counsel HIV-negative women in serodiscordant couples about the potential increased risk of HIV acquisition associated with progestogen-only injectable contraception, provide information on alternative contraceptive methods and health risks of unintended pregnancy and reinforce condom use and discuss availability of PrEP [42,43]. Following HIV-tailored FP counselling, women should be provided with comprehensive information on the effectiveness, side effects and follow-up required for the FP method of their choice. The contraceptive should be ideally provided within the HIV clinic, but if the service is not available or if the providers do not have the capacity to offer specific methods, such as long-acting or permanent contraception, the client should be supported with a facilitated referral to an appropriate facility.

### How should PCC services be offered?

Unfortunately, evidence on the best methods for providing PCC services to PLHIV in resource constrained settings is limited [8]. Whenever possible, full integration of PCC, HIV and FP services at the same location and at the same time, either in a healthcare facility or a community venue, is recommended to improve access to these services and to avoid missed opportunities for providing safer conception and FP services. Facilities, unable to offer the full range of services, should develop and implement a facilitated referral system. This includes active strategies for referring clients to required services, including patient escort, text message reminders, assisting clients to make appointments and case management approaches. Referral systems should also include a feedback mechanism to ensure that all referrals result in a received service and documentation of the referral and service received should be reflected in the client’s medical record.

Many countries experiencing both a high HIV burden and low modern contraceptive prevalence face a shortage of trained healthcare workers [44–46]. This shortage leads to long wait times and limited time for HIV or FP providers to offer comprehensive PCC services [8]. Task sharing, whereby certain tasks are moved to providers both in the medical and community setting with shorter training and fewer qualifications, is one strategy for addressing this challenge [47]. Lay counsellors, expert patients and community health workers can safely and effectively provide many of the PCC services outlined in Table 1 [47] and are also well positioned to provide follow-up support to PLHIV and their partners. Some countries in sub-Saharan Africa such as Uganda, Madagascar, Kenya and Ethiopia now allow trained community health workers to deliver FP methods directly to women [48]. Providing follow-up FP services in community settings can also help decongest overcrowded health facilities.

### Other considerations for implementing PCC services

Consideration of the social and structural context is critical when addressing preconception care for PLHIV and requires thoughtful planning. In many communities, men are involved in making decisions regarding future childbearing and FP use within the family; however, they are often not included in reproductive health discussions [49]. To address this gap, service implementation must provide an opportunity for shared counselling and FP decision-making. Men’s participation in pre-conception and antenatal care has been shown to improve uptake and continuation of a FP method [50–52], as well as improved uptake of and adherence to prevention of mother-to-child transmission (PMTCT) interventions [53,54], decreased infant HIV infection [55], decreased HIV transmission within serodiscordant couples [56], increased condom use [24,57] and reduced intimate partner violence [56]. Promising practices for male engagement in FP education and counselling include invitations from health providers for men to attend FP and PMTCT services [58,59], couple-based FP education [51,60], integration of FP and HIV services [61] and provision of services during non-working hours [62].

A specific challenge of preconception care for serodiscordant couples is the need to manage both the couple’s fertility goal and the potential HIV acquisition risk during the conception period, particularly in couples who have not disclosed their HIV status to each other. Evidence indicates that both men and women are reluctant to disclose their HIV status to their partner [63]. Thus, PCC programmes must include activities that include HIV status disclosure scenarios and notation in client medical records regarding disclosure status to partner. Whenever possible, programmes should encourage couples HIV testing and counselling with facilitated disclosure as part of PCC. Counsellors need to be attuned to the needs of their clients. For example, some clients may want their partner to know they are HIV positive but may not want to disclose how long they have been diagnosed. Counsellors can facilitate communication and couples can then consider options to reduce the risk of HIV transmission.

Stigma and discrimination by providers contributes to poor quality care [64] and is certainly not unique in consideration of PCC; however, there are often strong beliefs among providers regarding fertility intentions of PLHIV [65,66]. As a result, women and men may feel pressured to not report fertility intentions if they are HIV positive. Moreover, providers may be reluctant to counsel about FP methods other than condoms since they may believe that clients will reduce condom use. Activities must, therefore, address the misconceptions and resulting negative attitudes that some health providers hold regarding the fertility intentions and use of FP by PLHIV [67]. Health providers should be sensitized about the sexual and reproductive health rights of women, including PLHIV, and be trained to respectively discuss fertility intentions as well as FP and safer conception strategies with each client of reproductive age. This is especially important for adolescents and young women whose sexual and reproductive health needs are often not addressed within HIV care and treatment programmes and are often limited in FP settings [68].

Providing quality services requires an assessment of the health system capacity and competent and trained providers to offer PCC and FP services to PLHIV. Training providers in both the treatment and management of HIV as well as FP service provision is encouraged to facilitate integration of FP services within HIV clinical settings. National guidelines on HIV treatment and PMTCT services may need to be strengthened to include PCC and FP provision within HIV settings. Similarly, national guidelines on FP should include HIV testing, provision of PrEP and discussion of special considerations for PLHIV. These guidelines should include standard operating procedures and clinical tools for providing PCC and FP services to PLHIV and serodiscordant couples within HIV clinical care settings.

Evidence on how best to provide a PCC package in low resource settings is limited. While PCC is considered a foundation of the US national framework to eliminate paediatric infections, it is not common in resource-limited settings [69]. Clinical tools and programming frameworks that are in use in resource-limited settings should be replicated and scaled up across developing countries [70,71]. Further research is also needed to determine innovative service delivery models for providing PCC care and FP services to PLHIV. This includes determining who should deliver these services and where these services should be provided.

## Conclusions

Although much is known about the role of FP and PCC services in achieving safer conception, key challenges remain. Gaps include lack of national guidelines for integrating FP and PCC into HIV care, models for provision of quality services in low resource settings, provider stigma and discrimination, lack of male involvement and managing partner disclosure of HIV status. Research to test the replicability and scale up of PCC services, including provision of PrEP in FP and HIV services, should be prioritized to develop service delivery guidance that can be applied widely.

The USG continues to partner with local organizations, Ministries of Health, the private sector, civil society, multilateral and bilateral donors and other key stakeholders to strengthen both the policy and programme environment for safer conception and pregnancy counselling for PLHIV and serodiscordant couples. The USG remains committed to supporting and strengthening FP/HIV programming to increase PLHIV’s access to voluntary FP information and services, including safer conception counselling.
